# Perplexing Metabolomes in Fungal-Insect Trophic Interactions: A *Terra Incognita* of Mycobiocontrol Mechanisms

**DOI:** 10.3389/fmicb.2016.01678

**Published:** 2016-10-19

**Authors:** Digar Singh, Su Y. Son, Choong H. Lee

**Affiliations:** Department of Bioscience and Biotechnology, Konkuk UniversitySeoul, South Korea

**Keywords:** fungal interactions, entomotoxins, insect defense, metabolomes, metabotypes

## Abstract

The trophic interactions of entomopathogenic fungi in different ecological niches viz., soil, plants, or insect themselves are effectively regulated by their maneuvered metabolomes and the plethora of metabotypes. In this article, we discuss a holistic framework of co-evolutionary metabolomes and metabotypes to model the interactions of biocontrol fungi especially with mycosed insects. Conventionally, the studies involving fungal biocontrol mechanisms are reported in the context of much aggrandized fungal entomotoxins while the adaptive response mechanisms of host insects are relatively overlooked. The present review asserts that the selective pressure exerted among the competing or interacting species drives alterations in their overall metabolomes which ultimately implicates in corresponding metabotypes. Quintessentially, metabolomics offers a most generic and tractable model to assess the fungal-insect antagonism in terms of interaction biomarkers, biosynthetic pathway plasticity, and their co-evolutionary defense. The fungi chiefly rely on a battery of entomotoxins viz., secondary metabolites falling in the categories of NRP’s (non-ribosomal peptides), PK’s (polyketides), lysine derive alkaloids, and terpenoids. On the contrary, insects overcome mycosis through employing different layers of immunity manifested as altered metabotypes (phenoloxidase activity) and overall metabolomes viz., carbohydrates, lipids, fatty acids, amino acids, and eicosanoids. Here, we discuss the recent findings within conventional premise of fungal entomotoxicity and the evolution of truculent immune response among host insect. The metabolomic frameworks for fungal–insect interaction can potentially transmogrify our current comprehensions of biocontrol mechanisms to develop the hypervirulent biocontrol strains with least environmental concerns. Moreover, the interaction metabolomics (interactome) in complementation with other -omics cascades could further be applied to address the fundamental bottlenecks of adaptive co-evolution among biological species.

## Introduction

The kingdom of fungi has undergone critical metabolic advancements in defining its ecological interactions as an antagonistic or ammensalic, a commensalic, and a parasitic partner (**Box 1**) (Kempken and Rohlfs, 2010). The Inter- and intra- specific interactions in nature under the influence of various environmental factors are mediated through an array of metabotypes or metabolic phenotypes. These metabotypes, specifically the secondary metabolites, develop during the courses of ecological interactions of fungi and primarily belongs to polyketide, non-ribosomal peptides (NRP’s), alkaloid (lysine derived), and terpenoid classes (Sheridan et al., 2015). Biochemically, we can define these metabolic entities as “a heterogeneous class of low molecular weight compounds that, unlike primary metabolites, are not essential for the vital life functions i.e., growth or reproduction. However, these metabolites are extremely active at significantly low concentrations, and, can distinctly function as means of chemical communication or any sort of ecological interaction between fungi and its partners under a variety of environmental conditions (Karlovsky, 2008; Wiemann and Keller, 2014). Hence, an insight of the esoteric dynamic interplay of metabolic phenotypes and overall metabolomes under the ecological interface of fungal–insect interactions can provide an impetus to the ongoing efforts of developing the hypervirulent biocontrol strains with applications in sustainable agriculture, environment, and health through curbing the pest-borne diseases.

**Box 1. Glossary.**

**Antagonistic interactions:** the biotic interactions in ecosystem where one species is benefited at the cost of another interacting species. The antagonistic interactions are variously manifested as parasitism, predation, or antibiosis.

**Ammensalism:** ecological interaction between the organism of different species where one organism is inhibited or destroyed while the other remains unaffected.

**Commensalism:** the interaction among the members of two different species where one species is selectively benefitted whereas another one remains unaffected.

**Interactome:** the whole set of molecular interactions among the nucleic acids, proteins, or metabolites in a cell, tissue, organ, or an organism. The term is generally applied to the intra- or inter-molecular interaction of protein or proteinaceous entities.

**Metabolomes:** the collection of gross metabolites representing the current physiological state of an organism.

**Metabolomics:** it refers to the discipline involving the global evaluation of biochemical events in terms of the metabolite cues representing a particular physiological state of a cell, tissue, organ, or an organism.

**Metabotypes:** the metabolic phenotypes expressed or observed externally in response to intrinsic or environmental stimuli indicating a particular physiological state of an organism.

**Trophic interactions:** interaction among the organism at different levels of the food chain or food web.

Entomopathogenic fungi with nearly 750 species and 90 genera constitute the largest group of natural enemies to the pest insects nuisance to mankind in different ways. Most of the species from the classes, Zygomycetes and Ascomycetes, and Division – Amastigomycota, are known to be entomoparasitic (Roberts et al., 1991; Hajek, 1997; Roy et al., 2006; Molnár et al., 2010; Vega et al., 2012). Commercially, about 170 pest control agents are developed and marketed so far based on the 12 different entomopathogenic fungal species (de Faria and Wraight, 2007). The most pronounced species viz., *Metarhizum anisopliae* (Metsch.) Sorokin, *Beauveria bassiana, Vullemin, Isaria fumosorosea*, and *B. brongniartii* etc. (de Carolina Sánchez-Pérez et al., 2014), are well documented for the production of enzymes as well as chemically diverse and biologically potent entomotoxic metabolites.

## Entomopathogenic Fungi: Distributions

The entomopathogenic fungi, being the natural enemies to the umpteen varieties of insect and arachnid species exhibits a proportional cosmopolitan distribution. The entomopathogenic fungi are distributed to a myriad of habitats viz., soil, above or below ground plant parts, and host insect themselves in both aquatic as well as terrestrial environments.

### Soil

The soil being one of the most diverse environments for microbiological entities serves as the natural home for entomopathogenic fungal species. The different classes of biocontrol fungi have been documented from the soil (Keller et al., 2003; Meyling and Eilenberg, 2006). Functionally, the soil provides nutrients besides protection from the aerial anomalies like dehydration and harmful radiations. Soil usually shelters the fungal microflora under the suitable conditions of pH, humidity, and temperature (Keller and Zimmermann, 1989). The various soil conditions which primarily govern the distribution and density of entomopathogenic fungi includes geographical locations, climatic conditions, habitat type, cropping system, soil properties, and the numerous biotic as well as abiotic factors (Quesada-Moraga et al., 2007). The Inter- and intra-species chemical ecology of fungi is mainly regulated by accessibility to the nutrients and space, which governs their successful infection in host. The expression of fungal toxins and metabolites increase their ecological competitiveness to infect the corresponding host. The secretion of mycotoxin, zearalenone by *Fusarium* spp. helps the fungus to suppress the growth of competing species, and hence best reserve its host colonization conditions (Utermark and Karlovsky, 2007). The most renowned of the species includes, *Trichoderma* which produces a variety enzyme toxins viz., chitinases, glucanases, and proteases, all together helps it to compete best over their rival and host species making it most ubiquitous fungi in nature (Benítez et al., 2010). Moreover, a plethora of insects or plant hosts available in soil serves as the source of potential nutrients for fungi (Vega et al., 2009). Hence, soil ecosystem represents an amenable environment which facilitates the fungal species to fulfill its important ecological functions related with host mycoses and nutrition.

### Plants

A large number of biocontrol fungi are reportedly harbored by plants as endophytes or epiphytes (Arnold and Lewis, 2005). Here exist the synergistic interactions between the host plant and the fungi, which provide a defense shield to the host through its chemical weaponry of entomotoxic metabolites (Alabouvette et al., 2009). Plant-associated fungi are functionally classified as mycorrhizal, pathogenic, epiphytic, endophytic, and saprotrophic fungi (Porras-Alfaro and Bayman, 2011). The plant associated fungal species influence the chemical ecology of host plants toward the various biotic and abiotic stresses through either of the interactions viz., antagonism, parasitism, or mutualism effecting direct production of functional or elicitor metabolites. The most significant and well studied mechanism is the induction of “systemic acquired resistance (SAR)” mediated by plant stress metabolites viz., salicylic acid, jasmonic acid, ethylene, and a variety of pathogenesis-related (PR) proteins (Tripathi et al., 2008). The latent infection of maize varieties by *F. verticillioides*, producing mycotoxins viz., fumonisins, fusarins, and fusaric acids often positively regulates the yield and vegetative growth of the host plant. However, the increased production of fumonisin, owing to the altered abiotic or biotic conditions seldom cause infection of maize kernels resulting in the “*ear-rot*” disease in host (Glenn et al., 2008). The similar examples may include the species of *Beauveria, Lecanicillium*, and *Trichoderma*, which are best characterized to induce SAR in their respective host plants (Ownley et al., 2009).

### Host Insects

The entomopathogenic fungi enter and infect their target host through direct contact, making the former a more successful insecticide than their bacterial counterparts. The development of infection stages through conidia adhesion, penetration of insect cuticle by appresoria, and subsequent mycelia development are mediated through a range of hydrolytic enzymes viz., proteases, chitinases, lipases, and lipoxygenases (de Carolina Sánchez-Pérez et al., 2014). Once entered the insect host, the fungal mycelia grows as naked yeast-like propagules (blastospores), mechanically damaging the haemocoel and subsequently release a battery of entomotoxic metabolites. Although, the trophic interactions among the species are influenced by an infinite number of parameters of both biotic and abiotic origins, we would like to construe our interpretations in terms of the selected sets of metabotypes and altered metabolomes in fungal-insect antagonistic trophic interface (**Box 1**). The theoretical model of the present section of the review can best be visualized in Darwin’s famous exposition;

“*It follows that any being, if it vary, however, slightly in any manner profitable to itself, under the complex and sometimes varying conditions of life, will have a better chance of surviving, and thus be naturally selected.*”

(Chapter III – Struggle for existence, Origin of Species, Charles Darwin)

The majority of the well characterized insect associated fungi belongs to the order *Entomophthorales* (Phylum: *Glomeromycota*) and order *Hypocreales* (Phylum: *Ascomycota*), existing in both or either of their sexual (telomorph) or asexual (anamorph) phases of life cycle. The most significant of the insect associated fungi are isolated as anamorphs of the genus ‘*Cordyceps*’ viz., *Beauveria, Lecanicillium*, and *Isaria* (Blackwell, 2010). The commercial strains of *Metarhizium* and *Beauveria* are alone known to infect more than 200 species of different insect pests responsible for agricultural havocs (Toledo et al., 2008). The *in vivo* interactions between the entomopathogenic fungi and their insect host are antagonistic in nature, with hyperparasitic efficacy (Zimmermann, 2007; Vega et al., 2009). Here, both the pathogen (fungi) and the host (insect) evolves simultaneously in multiple dimensions viz., behaviorally, physiologically, ecologically, and metabolically to attain the necessary fitness to survive (Roy et al., 2006). These bizarre ecological relations are maintained with the help of a highly evolved biosynthetic machinery to produce the necessary mycotoxins which defines the trophic interactions of all entomopathogenic fungi. The chemistry of fungal interactions with insects is governed by a spectrum of cryptic metabolites falling into four major classes’ viz., NRP’s, alkaloids, terpenes, and polyketides (Rohlfs and Churchill, 2011). The major genera of domesticated entomopathogenic fungi (shown here using the classification system proposed by Alexopoulos and Mims, 1979), their insect host, related toxic metabolites, and commercial adaptations are summarized in **Table 1**.

**Table 1:** The major fungal divisions with entomopathogenic members, entomotoxic metabolites, and host insect range.

Taxonomic ranks↓	Entomotoxic metabolites	Host	Commercial formulations	Reference
Division: Amastigomycota (Non-flagellated terrestrial fungi)				
Subdivisions: ^∗∗^Ascomycotina (Telomorphs) and ^∗^Deuteromycotina (Anamorphs)				

***Genera***				

*Cordyceps*^∗∗^	Cordycepins	Lepidopteran larvae	–	Kim et al., 2002; Kryukov et al., 2014
*Hypocrella*^∗∗^/ *Aschersonia*^∗^	Ergosterol, Dustanin, Hypocrellins, 3-hopane-triterpenes	Aleyrodidae, Coccidae families of Hemiptera, and Nematodes	–	Isaka et al., 2003; Jin-Ming, 2006; Buttachon et al., 2013
*Beauveria*^∗^	Beauvericin, Bassianin, Oosporein, and bassianolide	Lepidoptera, Coleoptera, Hemiptera, Homoptera, and Hymenoptera	Naturalis^TM^, Botanigard^TM^, and Mycotrol O^TM^, Boverol, Brocaril, Ostrinil	Elsworth and Grove, 1977; Uma Devi et al., 2008
*Metarhizium*^∗^	Swainsonine, and Destruxins	Coleoptera, Hemiptera, Isoptera, Homoptera, Heteroptera, Diptera (Mosquitoes), Hymenoptera, Siphonaptera and Lepidoptera	MET52^TM^, Bioblast^TM^, BioPath^TM^, Green Guard ULV, and Green Muscle	Goettel et al., 2001; Quarles, 2013; Singh and Kaur, 2014a
*Paecilomyces*^∗^ (*Isaria*)	Beauvericin, Beauverolides, and Dipcolonic acid (DPA)	Hemiptera	PFR-97, PreFeRal, and Pae-Sin	Vey et al., 2001
*Verticillium*^∗^	hydroxycarboxylic acid, cyclosporine, and Dipicolonic acid, Bassianolide	Hemiptera and Thysanoptera (thrips)	Mycotal, Vertalec, and Bio-Catch	Vey et al., 2001
*Tolypocladium^∗^*	Efrapeptins, Tolypin, Diketopiperazines	Diptera (Mosquitoes), Ephemeroptera (Mayflies)	–	Bandani, 2004; Bandani, 2008
*Hirsutella*	Hirsutellin A and B	Mites (Citrus rust mites- *Phyllocoptruta oleivora*), Lepidotera (Galleria melonella)	Mycar	McCoy et al., 1992; Aghajanzadeh et al., 2006
*Nomuraea rileyi^∗^ (Cordycep^∗^)*	Ergosterol peroxide	Lepidoptera, Coleoptera, Hemiptera	AGO biocontrol nomuraea 50, PreFeRal	Prompiboon et al., 2008; Onofre et al., 2002
*Torrubiella*	Torrubiellin B (2)	Hemiptera (Coccoidea)	–	Isaka et al., 2012

Subdivision: Basidiomycotina				

***Genera***				

*Septobasidium*	–	Hemipteran scale insects –	Delicately mutualistic (often detrimental to insect spp.)	Hudson, 1986

Subdivision: Zygomycotina – specifically describing the newly classified members under the subdivision ‘Entomophthoromycotina’ as described by Humber (2012).				

***Genera***

*Entomophaga*	–	Orthoptera (grasshoppers), Coleoptera	–	Milner, 1997
*Erynia*	–	Hemiptera (aphids)	–	Milner, 1997; Pell et al., 2001
*Entomophthora*	–	Thysanoptera (thrips), Diptera (houseflies)	–	Pell et al., 2001
*Zoophthora*	–	Coleoptera, Diptera, Hemiptera, Hymenoptera, Lepidoptera, Orthoptera, Trichoptera	–	Glare and Milner, 1991; Pell et al., 2001

Division: Mastigomycota (Flagellated lower fungi)				
Subdivision: Haplomastigomycotina				

*Coelomycidium^∗^*	–	Dipterans (specially black flies)	–	Kim, 2011
*Myiophagus*	–	Dipterans	–	Karling, 1948; Araújo and Hughes, 2016

Subdivision: Diplomastigomycotina				

*Lagendium*	–	Dipterans (mosquito larvicidal)	Laginex AS, Laginex 25, LAGINEX^TM^	Kerwin et al., 1994; Hallmon et al., 2000; Vyas et al., 2007
*Leptogenia*	–	Dipterans (mosquito larvicidal)	–	Lastra et al., 2004; Pelizza et al., 2007, 2013
*Pythium*	–	Dipterans (Mosquito larvicidal)	–	Su et al., 2001

*The fungal classification system was primarily adapted from Alexopoulos and Mims (1979).*

## Antagonistic Metabolomes: Fungal–Insect Trophic Interactions

The active production of induced defense metabolites serves as a key defense mechanism against host insect’s counter immunity. Analogous antagonistic defense mechanisms are very common in plants, but are largely unexplored among fungi and their insect hosts. Here, we discuss the entomopathogenic fungi as the study model among their fungal counterparts. Most notable genera are the *Metarhizium, Beauveria*, and *Aspergilli* etc. which relies upon the polyketides, alkaloids, and NRP’s (non-ribosomal peptides) as their chemical shield or offensive tools of metabolites (Rohlfs and Obmann, 2009; Döll et al., 2013; Singh and Kaur, 2014a). Further, the *in vivo* expression of the toxin metabolite types and their relative quantities depends upon the respective insect host and numerous other factors (Skrobek et al., 2008). Either induced or intrinsic, expression of defense metabolites among the antagonistically interacting species triggers a state of metabolic plasticity.

### Fungal Metabotypes and Their Entomotoxicity Mechanisms

**FIGURE 1:**
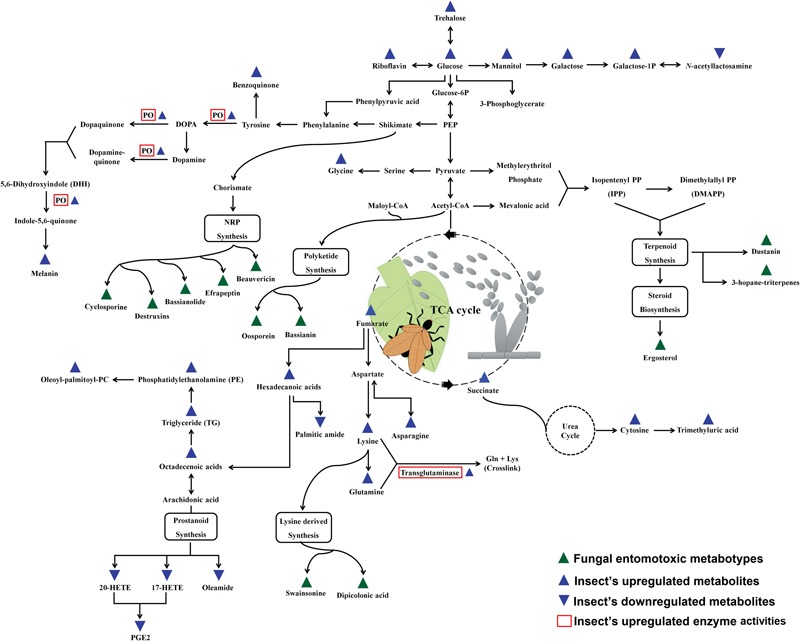
**Schematic representation of the fungal metabotypes and altered co-evolutionary insect metabolomes maneuvered during the antagonistic interactions.** The scheme of reference metabolic pathways is adapted from the KEGG (Kyoto Encyclopedia of Genes and Genomes) pathway maps.

**FIGURE 2:**
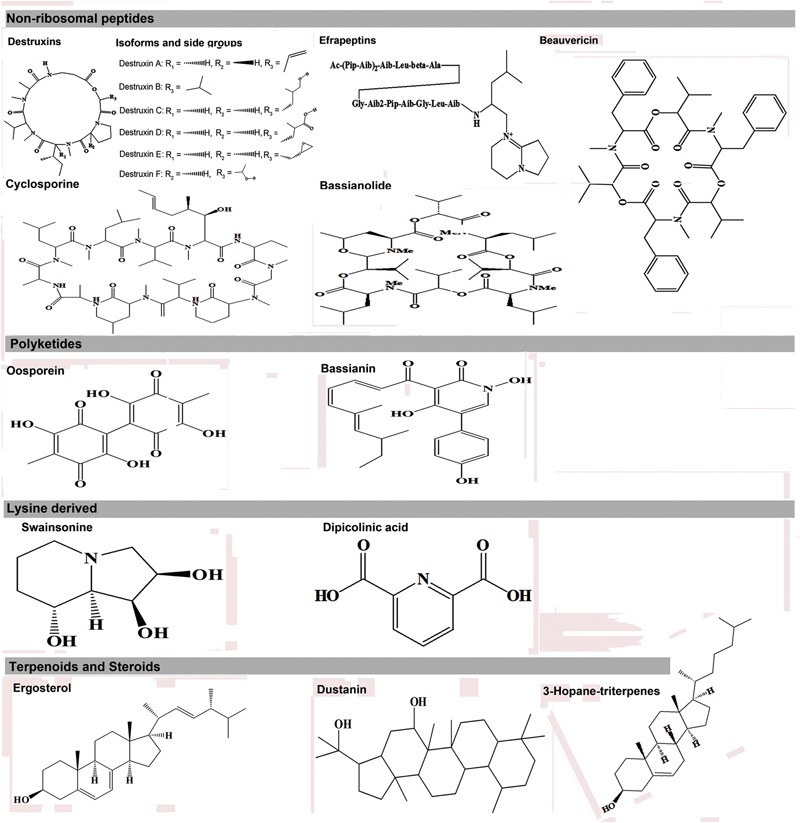
**Different entomotoxin structures and their respective biosynthetic classes in entomopathogenic fungi**.

The expression of metabolic phenotypes in fungi is a highly stringent process governed by the forces of natural selection ensuring its survival under altered ecological conditions. Although not necessary toward the major functions of growth and reproduction, secondary metabolites enable the fungi to survive and compete in an ecologically challenging environment viz., the presence of competing microorganisms, nutrient limitation, and protection against insect’s fungivory or evasion of host’s immune system (Wiemann and Keller, 2014). The fungal interactions with host insects drive their biosynthetic machinery to undergo altered metabolic states which we have envisaged using the Kyoto encyclopedia of genes and genomes (KEGG) pathway maps (Kanehisa and Goto, 2000), in **Figure 1**. Below, we introduce few of the umpteen metabotypes reportedly expressed during the stages of insect mycoses and briefly discuss their explicit entomotoxic effects. The chemical structures for these toxic fungal metabolites are shown in **Figure 2**.

### Non-Ribosomal Peptide (NRP’s)

#### Destruxins

The destruxins (Dxs) are structurally composed of an alpha-hydroxy acid and five amino acid residues joined together by amide and ester linkages to form a cyclic structure. There are 38 Destruxins or Dx analogs (Pedras et al., 2002), which is double the earlier reported, 19 types (Gupta et al., 1989). They are divided chemically into five basic groups labeled as A through E, plus several subgroups of each. Destruxins A and B were first reported in the *Metarhizium* isolates from Japan during 1960’s and were synonymously named as “oospora destructor” or destruxins. These cyclic depsipeptides have extensively been studied by plant pathologists, microbiologists, and natural products chemists for their toxic biochemical activities. The Dx – biosynthetic pathway is assumed to be a non-ribosomal multifunctional enzyme system (Kleinkauf and von Döhren, 1987, 1990; Turner, 2000). The insect specific toxicity of Dxs A, B, and E is well documented with reported induction of acute muscular paralysis in arthropods through the reversible opening of their muscle cell membrane Ca^+2^ channels (Samuels et al., 1988; Dumas et al., 1996). The ionophoric properties of Dx A also allow Ca^+2^ mobilizations across liposomal membrane barriers (Hinaje et al., 2002). Alternatively, Dxs are also reported to cause the inhibition of vacuolar (V-type) ATPase activity in the brush bordered midgut (BBM) membrane of *Galleria mellonella*, however, its structural stability is significantly compromised under the altered physiological conditions inside the host (Bandani et al., 2001). The Dx variants are further known for triggering the oxidative stress mechanisms in host (*Spodoptera litura*) through up regulating the levels of superoxide radicals and the systemic suppression of insect immunity (Pal et al., 2007; Sowjanya Sree et al., 2008). More recently, Meng et al. (2013) have described a yet another toxicity mechanism for Dx A on *Spodoptera litura* with deleterious effects on its wing disc like proteins (SLAWD) expressions, hence inducing the developmental abnormalities in larval stages of development.

#### Efrapeptins

These are the complex mixture of peptide toxins reported from the entomopathogenic soil hyphomycetes fungi, *Tolypocladium* spp. (Gupta et al., 1992). The variant form, Efrapeptin F is also known to significantly inhibit the activity of V-type ATPases, which regulates the protons gradient (K^+^/H^+^) across the brush bordered epithelium in insect mid-gut (Bandani et al., 2001). Simultaneously, the toxin has been reported to adversely affect the cell mediated immune mechanism in the host insect – *G. mellonella* (Bandani, 2008).

#### Beauvericin

It’s an ionophoric cyclohexadepsipeptide of enniatin antibiotic family which can permeate through biological membranes with enhanced entomotoxic activities (Gupta et al., 1991). Structurally, a beauvericin molecule is consists of the alternating amino acid units of the three D-hydroxyisovaleryl and three N-methylphenylalanyl residues (Hamill et al., 1969). The compound was first isolated from *B. bassiana*, but was later reported from *Fusarium, Verticillium*, and *Paecilomyces* spp. (Suzuki et al., 1977). The various diastereoisomers of beauvericin (A, B, and C etc.) shows the insecticidal activities against a spectrum of pest species viz., *Artemia salina, Calliphora erythrocephala, Aedes aegypti, Lygus, S. frugiperda*, and *Schizaphis graminum* (Grove and Pople, 1980; Jestoi, 2008; Wang and Xu, 2012). Functionally, the ionophoric nature of beauvericin allows the compound to form reversible complexes with divalent (Ca^+2^, Ba^+2^) and monovalent (Na^+1^, K^+1^) ions, which effectively disturb ionic equilibrium and pH potential across the lipid bilayers resulting in loss of membrane associated functions in insect host cells (Tang et al., 2005; Tonshin et al., 2010).

#### Bassianolides

The bassianolides are structurally similar to beauvericin except the alternate four subunit repeats of d-2-hydroxyisovaleric acid (D-α-Hiv) and N-methyl Leucine (L-*N*-Me-Leu) in the cyclooligomer depsipeptide COD making it an octadepsipeptide (Suzuki et al., 1977). As an entomotoxic COD, bassianolide from *B. bassiana* and *V. lecanii* is known for inducing acute muscular atony among silkworm larvae through the inhibition of acetyl-choline mediated muscle contractions (Kanaoka et al., 1978; Nakajyo et al., 1983). Few of the reports have suggested the predominant role of bassianolides in fungal virulence as compared to other cyclodepsipeptides i.e., beauvericin, (Champlin and Grula, 1979; Xu et al., 2009). The high efficacy of these compounds as an effective entomotoxin can also be attributed to its structural conformation with hydrophobic exterior and relatively hydrophilic interior making it an ionophoric molecule. A wide range of pest species are reportedly affected through bassianolide exposure viz., *Helicoverpa zea* (Champlin and Grula, 1979), chagas vector *Triatoma infestans* (Lobo et al., 2015), and livestock pest *Culicoides* spp. (Narladkar et al., 2015) etc.

#### Cyclosporines

Typically known for their immunosuppressive application for organ transplant, cyclosporines were originally reported from *Tolypocladium niveum*, and more recently from *T. inflatum* (Weiser and Matha, 1988; Bushley et al., 2013). Specifically, cyclosporine A is known to suppress the insect’s humoral or innate immune responses (Fiolka, 2008; Kulkarni et al., 2013). Earlier, the potential suppression of cyclosporine sensitive glycoprotein based eﬄux pump system in insect cells was recognized as the probable mechanism of insect mycoses (Podsiadlowski et al., 1998). Concomitantly, Fiolka (2008) have further proposed an alternate mechanism of cyclosporine mediated entomoxicity i.e., decrease in the activity of insect’s antimicrobial peptides and lysozymes which adversely effects its survival.

### Polyketide (PKs)

#### Oosporein

A non-reduced polyketide commonly reported from *Beauveria* spp. (Vining et al., 1962; Strasser et al., 2000). Recently, Feng et al. (2015) have verified the role of oosporein (bibenzoquinone oosporein) in establishing the fungal virulence in host insects through the inhibition of insect defense mechanisms viz., PPO (pro-phenoloxidase) activity and down-regulation of antifungal peptide expressions in host.

#### Bassianin

It represents a hexaketide compound with a 2-pyridone core reported first from *Beauveria* spp. and associated with a broad range of biological activities (McInnes et al., 1974). Alternatively, the compound has been reported to inhibit the Ca^+2^ dependent ATP’ase activities in mammalian erythrocytes (Jeffs and Khachatourians, 1997). However, no elaborated reports are yet available which discuss the specific entomotoxic effects associated with bassianin.

### Lysine Derived

#### Swainsonine

Swainsonine is chemically an indolizidine alkaloid molecule with a fused piperidine and pyrrolidine ring system. This sugar analog was first discovered in Australian native legume *Swainsona canescens* (Colegate et al., 1979) followed by *Astragalus* and *Oxytropis* (Molyneux and James, 1982) as a toxin metabolite responsible for locoweed poisoning among livestock. Later on, the compound was also reported from microbial sources viz., *Rhizoctonia leguminicola* (Schneider et al., 1983) and *M. anisopliae* (Hino et al., 1985). Functionally, swainsonine acts as the reversible inhibitor of both lysosomal α-mannosidase and mannosidase II enzymes which mainly catalyze the cellular degradation of polysaccharides and asparagine-linked glycoproteins, respectively (Elbein et al., 1981). These properties of swainsonine have been well documented and maneuvered to develop the anti-metastatic and anti-proliferative candidates through laboratory based and clinical trial studies (Sun et al., 2009; Li et al., 2012; Singh and Kaur, 2014b). Although many studies have described the therapeutic potentials of swainsonine, its entomotoxic properties and role in the entomopathogenic virulence of *Metarhizium* are still largely unexplored. Recently, Singh and Kaur (2014a) have reported the *in vitro* entomotoxic properties of swainsonine isolated from *M. anisopliae* against the lepidopteran target host (*Spodoptera* sp.) through the induction of apoptotic cell death mechanisms. However, the potential role of swainsonine as an entomotoxin for establishing the fungal – insect chemical ecology requires further studies.

#### Dipicolinic Acid

The potent entomotoxic metabolite is variously been reported from numerous entomopathogenic genera viz., *Beauveria, Paecilomyces*, and *Verticillum* (Claydon and Grove, 1982). The pyridine derivative compound i.e., dipicolinic acid (DPA) or pyridine-2, 6-dicarboxylic acid is ubiquitously found in all bacterial spores and confers them thermal resistance (Setlow et al., 2006). In the context of entomotoxicity, the fungal DPA or their calcium salts are reportedly known to be active against white fly larvae (*Bemisia*) and blowflies (*C. erythrocephala*) with varying degrees of toxicity (Asaff et al., 2005).

### Terpenoids and Steroids

The potent entomotoxic effects for fungal terpenoid and steroid metabolites can be correlated analogously with those of plant steroid where these metabolites serve as juvenile hormones which alter the development and behavior of herbivore insects or seldom induce direct toxicity (Schardl, 2001). The crude extracts from acaricidal fungi *Hypocrella raciborskii* were characterized for terpenoid and steroid metabolites viz., ergosterol, dustanin and 3β-acetoxy-15α,22-dihydroxyhopane (a hopanoid) with varying mechanism of insect deterrence and toxicity (Buttachon et al., 2013). Further, Isaka et al. (2010) have reported the new terpene compounds from entomopathogenic fungi *Aschersonia paraphysata* with potential *in vitro* anti-malarial activity for selected hopene metabolites i.e., 17(21)-hopene-6R,12β-diol. Hence, one can summarize the antagonistic interactions for terpenoid and steroid class of entomotoxic metabolites which potentially facilitate in insect mycoses or deter the fungivory.

An imponderable number of fungal metabolites acting as entomotoxins ensure their successful ecological succession to overcome the antagonistic arthropod hosts. The fungi, like any other organism are also subjected to the incessant process of natural selection under different environmental conditions including the host’s trophic interfaces with antagonistic metabolomes.

## Insect’s Co-Evolutionary Metabotypes and Metabolome

The host insects too have developed the competitive co-evolutionary defense mechanisms to survive the proportional selection pressure from their respective mycoparasites. The three main lines of insect defense i.e. cuticular, humoral, and cellular responses together resist the entry of fungal pathogens (Dubovskiy et al., 2013). The first and the most vital of the barrier is cuticular, which prevent the entry of fungal infectious forms i.e., conidia or blastospores, releasing a battery of entomotoxic metabolites and hydrolytic enzymes viz., proteases, chitinases, and lipases (Xiao et al., 2012; Ortiz-Urquiza and Keyhani, 2013).

### Insect Defense Metabotypes

Once the fungal appresoria breach through the insect cuticle, the enhanced antiproteolytic and phagocytic activities of insect hemolyph and plasmatocytes, respectively, prevents the further stages of fungal mycoses (Boguś et al., 2007). Besides, the cuticular secretions with fungal enzyme inhibitors and increased phenoloxidase activity (humoral components) are also reported to impede the progression of mycoses (Dubovskiy et al., 2013). However, the role of host insect metabolites is seldom considered important during the different stages of microbial infection and thus remains largely unexplored. Recently, Pedrini et al. (2015) have reported the benzoquinone containing secretions in tenebrionid insects (*Tribolium castaneum*) as the defensive means against the infestation from entomopathogenic fungi (*B. bassiana*). The arthropodal quinones can potentially impair the invading pathogens through a number of non-specific defensive phenomena viz., production of ROS (reactive oxygen species) and melanin cross linking of infectious bodies (Nappi et al., 2009). As shown in **Figure 1**, the phenoloxidase (PO) activities in arthropods effectively maneuvers the phenylalanine (Phe) conversion to tyrosine and a number of biosynthetic pathway intermediates viz., 3,4 dihydroxyphenylalanine (DOPA), Dopamine, and quinone derivatives before finally been converted to melanin compounds (Vilmos and Kurucz, 1998; Fuchs et al., 2014). The upregulation of insect PO activities and melanin biosynthesis effectively cripples the fungal infections inside the host insect through the effective deposition and hardening around the hemocyte encapsulated infectious bodies i.e., blastospores (Smilanich et al., 2009). Hence PO activity simultaneously induced both humoral as well as cellular immune response in arthropods. The melanic strains of *G. mellonella* (greater wax moth) are known for their heightened resistance to *Metarhizium* or *Beuveria* induced mycoses on account of their up-regulated PO activity and thickened deposition of cuticular melanin (Dubovskiy et al., 2013). Hence, the Pro-PO mediated PO-cascade can undoubtedly be considered as an essential component of insect’s innate immunity and thus is tightly regulated by a series of enzyme competitive complexes and signaling pathways i.e., Toll signaling pathways (Kan et al., 2008). Quintessentially, the host-parasite selective co-evolution has resulted in a state of competitive coercion for ecological fitness and survival.

### Altered Metabolomes and Insect’s Immune Response

#### Carbohydrates, Lipids, and Fatty Acids

Yet another aspect of insect immunity can be framed using the concept of “*immunity bioenergetics’* under the condition of insect mycoses. The estimated cost of immune response in vertebrates is variedly calculated approximately 32%, thus, by analogy, one can assume the respective metabolic expenditures in case of insect mycoses or infections (Martin et al., 2008). The humoral (PO-mediated) and cellular (hemocyte mediated) immunity in infected insects entails bioenergetic cost among the host which can be tracked in its metabolome. A recent progress has led to the identification of the metabolic cues associated with insect’s altered metabolomic response to the co-evolutionary selection pressure by entomopathogenic fungi. Xu et al. (2015) have reported the alteration in the levels of energy and nutrient metabolism of silkworm moths infected with *B. bassiana*. As indicated in **Figure 1**, the group has observed an upregulation in the levels of carbohydrates, fatty acids, lipids, and amino acids with simultaneous down regulation in eicosanoids and amines. The proposed elevation in the amounts of carbohydrates (sugars) was correlated to the increased ratio of monosaccharide to disaccharide sugars owing to possible bio-conversion to meet the heightened energy cost for immunity and metabolism (Ardia et al., 2012). Another vital component of insect immune response tractable in its metabolome is comprised of lipids, fatty acids, and eicosanoids i.e., prostanoids. Intriguingly, the levels of various lipids viz., Phosphatidylethanolamine, triglycerides, glycerophosphocholine, and 1-oleoyl-2-palmitoylphosphocholine are reportedly increase in case of both the fungal as well as bacterial infections of host insects (Hoxmeier et al., 2015; Xu et al., 2015). The atypical elevations of the lipid levels thus fulfill the heightened biomolecular demands for energy generations, membrane repair, and signaling pathway intermediates (Atella and Shahabuddin, 2002). Hence, the alterations in the lipid levels may also serve as the potent and generic biomarkers of insect immunity under immune compromised conditions. Similarly, the fatty acids released from triglycerides viz., hexadecenoic acid, heptadecenoic acid, and octadecenoic acid further fulfill the energy demands through β-oxidation (Athenstaedt and Daum, 2006).

#### Amino Acids

The upregulation of amino acid metabolism (asparagine, glutamine, lysine) and transglutaminase activity (amino acid cross links forming target clots) are often corresponded to the enhanced humoral immune response among the infected arthropods (**Figure 1**) (Bulet et al., 1991; Toubarro et al., 2013). In contrast, the elevation in the levels of the free amino acids in hemocoel might also be attributed to the proteolytic activities of the invading microbial parasites (Harrison and Bonning, 2010). An *avant-garde* experiment published by Graham et al. (2014) describes the different diet preferences of locusts (*Chortoicetes terminifera*) subjected to *Metarhizium* infection *in vitro*. The authors observed that the locust which switched their feeding to high carbohydrate diets survived the fungal infection more effectively than their counterparts fed upon high protein diets. Hence, a logical conjecture was drawn that the entomopathogenic fungi can more efficiently harness the protein contents from the insect hemocoel than the host themselves, and thus the high mortality was observed among the protein rich diet fed insects. Therefore, the meticulous and more robust metabolomic experimental design is required to differentiate the free amino acids and related metabotypes in hemocoel characterized for their origin while the stages of mycosis.

#### Eicosanoids

They are the signaling metabolites produced from the oxygenated poly-unsaturated fatty acids and functionally important for the immune responses in insects (Stanley, 2006). In particular, the insects infected with entomopathogenic fungi are reportedly known to have the reduced levels of eicosanoids viz., 17-hydroxyeicosatetraenoic acid (17-HETE) and protaglandins - E2 (PGE2) (Xu et al., 2015). The mechanism seems more pertinent as the suppression of eicosanoids and prostanoids is analogously reported in case of entomopathogenic bacteria and nematode infections thus establishing their vital role in the insect’s innate immunity (Hyrsl et al., 2010; Hwang et al., 2013; Hoxmeier et al., 2015).

Additionally, the reduced host immunity can further be implicated based on the upregulation of cytosine (nitrogenous bases) and trimethyluric acid, a purine alkaloid (Xu et al., 2015). Further, Diaz-Albiter et al. (2012) have suggested the deleterious effects of uric acid components on insect’s ROS mediated immunity, though the phenomena is quite unclear in case of *in vivo* metabolome alterations following mycoses. Nonetheless, there are many more esoteric facets of insect immunity besides the intrinsic immune response and metabolomes which needs to be delineated (**Box 2**). Quintessentially, the relative selection pressure induced by the host-pathogen interaction can thus be called as the main driving force behind the altered insect immunity and adaptation in the challenged environments.

**Box 2. The Gordian knot: biocontrol fungi and host insect’s interaction metabolomics.**

The metabolites and metabolomes being the most generic cues governing the biological interactions are undoubtedly the most critical factors which govern fungal biocontrol mechanisms. However, our present comprehensions are surprisingly limited regarding perplexes of host insect’s co-evolutionary metabolomes and the role of environmental factors in shaping these interactions. The varying efficacy of broad range entomopathogenic fungi toward insects of similar classes further compels us to re-examine our nebular hypothesis regarding the mechanisms of insect mycoses. The modern assumptions credit this differential effectiveness of entomopathogenic fungi to the host insect’s co-evolution in the challenged environments through co-interactions with symbiotic microflora which passively confer a protective chemical shield of anti-fungal metabolites or enhanced immune response (Toledo et al., 2011; Eleftherianos et al., 2013). However, it is still unclear about the chemical nature of these metabolites, either of fungal or insect origin, which actually instigates the chain of conflicting events among the interacting species. Additionally, we don’t know how these elicitor molecules (metabolites) affect the priming of co-evolutionary multi-trophic interactions among them at genomic platforms? Nonetheless, it can only be assumed that the scarce data and information available thus far represent the tip of the iceberg with limited comprehensions for biocontrol sciences and fungal trophic interactions.

## Conclusion

The evolution of recalcitrant pest varieties and increased environmental concerns owing to the use of synthetic chemical pesticides has turned the attempts toward the development of efficacious biopesticides, a non-trivial undertaking. However, the relative progress in the development of efficient biopesticides and their ground formulation are apparently stonewalled on account of our surprisingly limited comprehension of the quasi simplistic biocontrol mechanism. In recent years, a renewed interest has grown among the researchers to delineate the biocontrol mechanisms in more unconventional ways viz., target pest behavior (Shang et al., 2015), *de novo* genome assemblies for pathotype characterizations (Shang et al., 2016), host-pathogens interaction transcriptomics (Chu et al., 2016), and metabolomics (de Bekker et al., 2013; Xu et al., 2015) etc. Hence, the trophic interactions of the ubiquitously distributed entomopathogenic fungi in diverse environmental habitats can further be envisaged in terms of their altered metabolomes which offers a generic harbinger to address the key bottlenecks of associated biocontrol mechanisms. The trophic interface between entomopathogenic fungi and corresponding host insects has often been construed for fungal entomotoxins which impairs their targets. However, the proportional immune response conferring immunity in host insects can also be extrapolated for altered metabolism and defense biomarkers viz., PO-mediated melanin synthesis, insect bioenergetics (carbohydrates, fatty acids, and lipid), free amino acids, antimicrobial peptides, and eicosanoids. Additionally, the regulatory networks and signal transduction pathways affected in mycosed host insects could also be probed and correlated with fungi mediated selective perturbations. Hence, a metabolomic insight of the fungal–insect antagonistic interactions could potentially reshape our current strategies to develop the selective, broad target, and hypervirulent entomopathogenic fungal strains. Besides the realms of much touted biocontrol applications, the new facets of entomopathogenic fungi interactions as plant endophyte, rhizopheric colonizer, and soil inhabitant can also be addressed using the newfangled omic-approaches.

## Author Contributions

DS, SS, and CL have made conceptual as well as direct contribution in writing this manuscript.

## Conflict of Interest Statement

The authors declare that the research was conducted in the absence of any commercial or financial relationships that could be construed as a potential conflict of interest.
